# Division of cortical cells is regulated by auxin in *Arabidopsis* roots

**DOI:** 10.3389/fpls.2022.953225

**Published:** 2022-09-14

**Authors:** Huijin Kim, Jinwoo Jang, Subhin Seomun, Youngdae Yoon, Geupil Jang

**Affiliations:** ^1^School of Biological Sciences and Technology, Chonnam National University, Gwangju, South Korea; ^2^Department of Environmental Health Science, Konkuk University, Seoul, South Korea

**Keywords:** auxin, cortex, cortical cell division, polar auxin transport, epidermal cell fate, *Arabidopsis*

## Abstract

The root cortex transports water and nutrients absorbed by the root epidermis into the vasculature and stores substances such as starch, resins, and essential oils. The cortical cells are also deeply involved in determining epidermal cell fate. In *Arabidopsis thaliana* roots, the cortex is composed of a single cell layer generated by a single round of periclinal division of the cortex/endodermis initials. To further explore cortex development, we traced the development of the cortex by counting cortical cells. Unlike vascular cells, whose number increased during the development of root apical meristem (RAM), the number of cortical cells did not change, indicating that cortical cells do not divide during RAM development. However, auxin-induced cortical cell division, and this finding was confirmed by treatment with the auxin transport inhibitor *N*-1-naphthylphthalamic acid (NPA) and examining transgenic plants harboring *CO2::ΔARF5*, in which cortical expression of truncated AUXIN RESPONSE FACTOR5 (*ΔARF5*) induces auxin responses. NPA-induced cortical auxin accumulation and *CO2::ΔARF5-*mediated cortical auxin response induced anticlinal and periclinal cell divisions, thus increasing the number of cortical cells. These findings reveal a tight link between auxin and cortical cell division, suggesting that auxin is a key player in determining root cortical cell division.

## Introduction

The plant root contains a variety of tissues with specialized functions. The root cortex, which is located between the root epidermis and endodermis, mediates the transport of water and nutrients absorbed by the epidermis into the central vascular tissue *via* apoplastic and symplastic routes. The root cortex is also responsible for storing photoassimilates in the form of starch, which is imported from the shoot through the phloem, and a variety of secondary metabolites including resins, latex, essential oils, and tannins (Von Fircks and Sennerby-Forsse, 1998; Lux et al., 2004; Azhar et al., 2019).

In *Arabidopsis thaliana* roots, the cortex is part of the ground tissues and originates from the cortex/endodermis initial (CEI) in the stem cell niche. The CEI produces two types of cells *via* a single round of periclinal division; the resulting cells develop into cortical and endodermal cells. Consequently, this developmental process forms double layers of ground tissues, which contain a single layer of cortex and endodermis (Benfey et al., 1993; Dolan et al., 1993; Scheres et al., 1994). Several key genes have been identified that are related to the asymmetric periclinal division of the CEI and the formation of the cortex initial cell, such as *SHORT-ROOT* (*SHR*) and *SCARECROW* (*SCR*). SHR is a key regulator of root growth and development. The movement of stele-expressed SHR proteins to the CEI activates the expression of *SCR* and triggers the asymmetric periclinal division of the CEI (Nakajima et al., 2001; Gallagher et al., 2004). The essential roles of SHR and SCR in this process are supported by the phenotypes of *SHR* and *SCR* knock-out plants. In *shr* and *scr* mutants, the asymmetric periclinal division of the CEI does not occur, and only a single layer of ground tissue called the mutant layer is formed (Benfey et al., 1993; Scheres et al., 1995; Di Laurenzio et al., 1996; Helariutta et al., 2000). Although these findings explain the initial formation of cortical cells in the stem cell niche, the subsequent steps of cortical cell development remain largely unknown.

Auxin is a key phytohormone that regulates all aspects of plant development and growth. Auxin induces an auxin response in cells *via* an auxin-specific signaling pathway involving AUXIN RESPONSE FACTORs (ARFs). These key transcription factors, which are responsible for the mass transcription of auxin-responsive genes, typically consist of four domains: a DNA-binding domain, an activation domain, and domains III and IV (Ulmasov et al., 1999; Tiwari et al., 2003; Li et al., 2016). Domains III and IV mediate the interactions between the ARFs and the auxin signaling repressor proteins Aux/IAAs. These interactions suppress the cellular auxin response by inactivating the transcriptional activities of ARFs. In response to cellular auxin, the interaction between ARFs and Aux/IAAs is abolished by *SCF*^TIR1^ E3 ligase-mediated proteolysis of the Aux/IAAs, leading to release of the ARFs and the activation of the auxin response (Gray et al., 2001; Weijers et al., 2005; Mockaitis and Estelle, 2008).

The essential role of ARFs in auxin responses has been confirmed *via* many genetic and molecular biological studies. For example, gain-of-function *axr3-1* mutants produce a mutant version of Aux/IAA17 with a single amino acid change, and have severely compromised auxin responses. Because the mutant Aux/IAA17 protein is resistant to *SCF*^TIR1^ E3 ligase-mediated degradation, the interaction between this protein and ARFs is maintained and ARFs are inactivated, regardless of cellular auxin levels (Leyser et al., 1996; Rouse et al., 1998; Bishopp et al., 2011). Also, a knock-out mutation of *ARF5* suppresses the auxin response, whereas overexpression of a truncated form of ARF5 that is unable to interact with Aux/IAAs is sufficient to activate the auxin response (Hardtke and Berleth, 1998; Liscum and Reed, 2002; Krogan et al., 2012).

The auxin response in roots is position-dependent. This position-dependent auxin response regulates root cell and tissue development by providing key information required for cell fate determination, cell division and differentiation, and polar auxin transport, representing a key mechanism governing position-dependent auxin accumulation and auxin-dependent development (Friml et al., 2002; Teale et al., 2006; Vanneste and Friml, 2009). Polar auxin transport also mediates the establishment of directional auxin flow, including acropetal (toward the root tip through the central vasculature) and basipetal (toward the root base through the epidermis) flow (Rashotte et al., 2000; Muday and DeLong, 2001; Friml et al., 2002; Friml, 2003; Blilou et al., 2005; Michniewicz et al., 2007; van Berkel et al., 2013). Acropetal and basipetal auxin flow are thought to be linked to centripetal auxin flow, which transports auxin from the epidermis to the central vasculature (Kepinski and Leyser, 2005; Lewis et al., 2007; Baluška et al., 2010).

In this study, we investigated the development of the cortex in *Arabidopsis* roots. Unlike vascular cells, the number of cortical cells did not change during root development, indicating that root cortical cells that initially form by the asymmetric periclinal division of the CEI do not further divide. However, cortical auxin accumulation *via* the treatment of an auxin transport inhibitor strongly activated the division of cortical cells, suggesting that auxin induces cortical cell division. This notion was supported by the finding that cortical cell division was promoted in *CO2::ΔARF5* transgenic plants whose cortical auxin response was activated by the cortical-specific expression of truncated *ARF5* (*ΔARF5*). Collectively, these results suggest that auxin is a key regulator of the division of cortical cells in *Arabidopsis* roots.

## Materials and methods

### Plant materials and growth condition

*Arabidopsis thaliana* ecotype Columbia-0 was used as the wild-type control. The *DR5::VENUS* line was described previously (Brunoud et al., 2012; Seo et al., 2021). Seeds were obtained from the Nottingham Arabidopsis Stock Centre. Seeds were sterilized for 15 min in 70% ethanol and then for 15 min in 100% ethanol, and sown on 1/2 Murashige and Skoog (MS) solid medium. After vernalization at 4°C for 4 days in the dark, plants were grown vertically at 23°C under a 16/8 h (light/dark) cycle. For auxin and *N*-1-naphthylphthalamic acid (NPA) treatment, plants were grown on 1/2 MS solid medium supplied with 500 nM indole-3-acetic acid (IAA) and 3 μM NPA for 7 days.

### Construction of *CO2::ΔARF5* and *IAA2::GFP* plasmids and plant transformation

To construct the *CO2::ΔARF5* plasmid, a DNA fragment containing the *CO2* promoter, which exhibits cortex-specific expression (Heidstra et al., 2004), was amplified by PCR. The DNA fragments were inserted into the HindIII/KpnI-digested pMDC plant binary vector using the Gibson Assembly Cloning system (New England BioLabs). To generate ΔARF5 cDNA, total RNA was extracted from 7-day-old *Arabidopsis* plants grown on 1/2 MS solid medium using RNeasy Plant Mini-prep kits (Qiagen). Reverse transcription was performed using 1 μg of total RNA, oligo dT primers, and Superscript III reverse transcriptase (Invitrogen). The ΔARF5 cDNA was amplified by PCR. The ΔARF5 cDNA fragment was introduced into the pDONR221 vector by BP reaction using the GATEWAY system (Invitrogen). The ΔARF5 cDNA fragment in the pDONR vector was inserted into the *CO2* promoter-carrying pMDC plant binary vector using LR Clonase (Invitrogen). For the construction of *IAA2::GFP* plasmid, *IAA2* promoter which induces xylem-specific transcription (Bishopp et al., 2011) was amplified by PCR, and then inserted into pDONR P4-P1R *via* a BP reaction. The IAA2 promoter was inserted into the GFP-carrying dpGreen binary vector using MULTISITE GATEWAY LR recombination system (Invitrogen). To generate transgenic *Arabidopsis* expressing *CO2::ΔARF5* or *IAA2::GFP*, the recombinant plasmids were introduced into Col-0 wild-type using the floral dip method, as described previously (Clough and Bent, 1998). Primer sequence information for the construction is available in Supplementary Table 1.

### Embedding, sectioning, and staining

To visualize the internal anatomy of *Arabidopsis* roots, physical sectioning was performed using the Technovit 8100 system (Heraeus Kulzer, Hanau). To embed the *Arabidopsis* roots, the samples were fixed in 4% paraformaldehyde for 1 h, washed five times with ddH_2_O, and incubated overnight at room temperature. The roots were realigned on a 1 mm layer of solid 1.2% agarose and covered with 1.2% molten agarose. After solidification, the samples were cut into small blocks, washed three times in ddH_2_O for 30 min, and dehydrated in a graded ethanol series (25, 50, 75, and 100% [v/v] in ddH_2_O), 1 h per step. The dehydrated samples were incubated in a Technovit 8100 series (25, 50, 75, and 100% [v/v] in EtOH) for 1 h per step, incubated in 100% Technovit 8100 solution for 1 day, and placed in molds. To solidify the samples, a mixture of Technovit 8100 and hardener solution (15:1 [v/v]) was added to the molds, and the samples were incubated at room temperature for 2 days. Sections (3–3.5 μm) were taken from the apical meristem and maturation regions of the roots using a Leica RM 2145 sectioning machine. The sections were stained with 0.05% toluidine blue solution for 1.5 min and washed with ddH_2_O.

### RT-qPCR analysis

RT-qPCR analyses were performed using total RNA extracted from the NPA-untreated and treated roots. To synthesize the first-strand cDNA, reverse transcription was performed using 1 μg of total RNA and Superscript III reverse transcriptase (Invitrogen). For qPCR, a master mix was prepared using a LightCycler 480 SYBR GREEN I Master (Roche). PCR reactions and fluorescence detection were performed using a LightCycler NANO Real-Time PCR machine (Roche). PCR conditions were programmed according to the manufacturer’s instructions (initial denaturation at 95°C for 5 min, denaturation at 95°C for 10 s, annealing at 60°C for 10 s, and extension at 72°C for 10 s for 45 cycles). Three technical replicates of the qRT-qPCR were performed using three biological replicates. *ACTIN2* (*ACT2*) was used as an internal control. Primer sequence information is available in Supplementary Table 1.

### Microscopic observations

To visualize fluorescent signals in wild-type Col-0, *CO2::ΔARF5*, and *DR5::VENUS*, whole roots of the indicated plants grown in 1/2 MS medium for 1 week were dipped in propidium iodide (PI) solution (10 μg/ml) for 2 min. After staining, the roots were mounted on glass slides in ddH_2_O. For excitation, 561 nm light for PI fluorescence and 488 nm light for VENUS fluorescence were used. Fluorescence was visualized at wavelengths of 591–635 nm for PI and 505–530 nm for VENUS. The fluorescent signals were visualized and captured under a Leica HCS A confocal microscope (Leica HCS A), and Z-stack images were obtained using Leica LAS X software. To visualize cortex development and quantify anticlinal and periclinal division of cortical cells in the indicated plants, root sections were examined with Leica DM 2500 light microscope, and images were captured with a Leica DFC450 C camera mounted on the light microscope. The frequency of anticlinal and periclinal division of cortical cells was obtained by dividing the number of plants with anticlinal and periclinal division by the total number of plants we tested (*n* > 15). To visualize root hair patterning and quantify root hair abnormality, the samples were examined with Zeiss Discovery.V12 stereo microscope, and images were captured with Axiocam 105 color camera mounted on the stereo microscope. The root hair abnormality was obtained by dividing the number of plants with the double H cell file phenotype by the total number of plants we tested (*n* > 20).

### Statistical analysis

Data were presented as mean values, and the number of samples is indicated in the figure legends. The statistical difference between the samples and their control was determined using a two-tailed Student’s *t-*test with a *p* < 0.01.

## Results

### Cortical cell division is not coordinated with root apical meristem cell division

**Figure 1:**
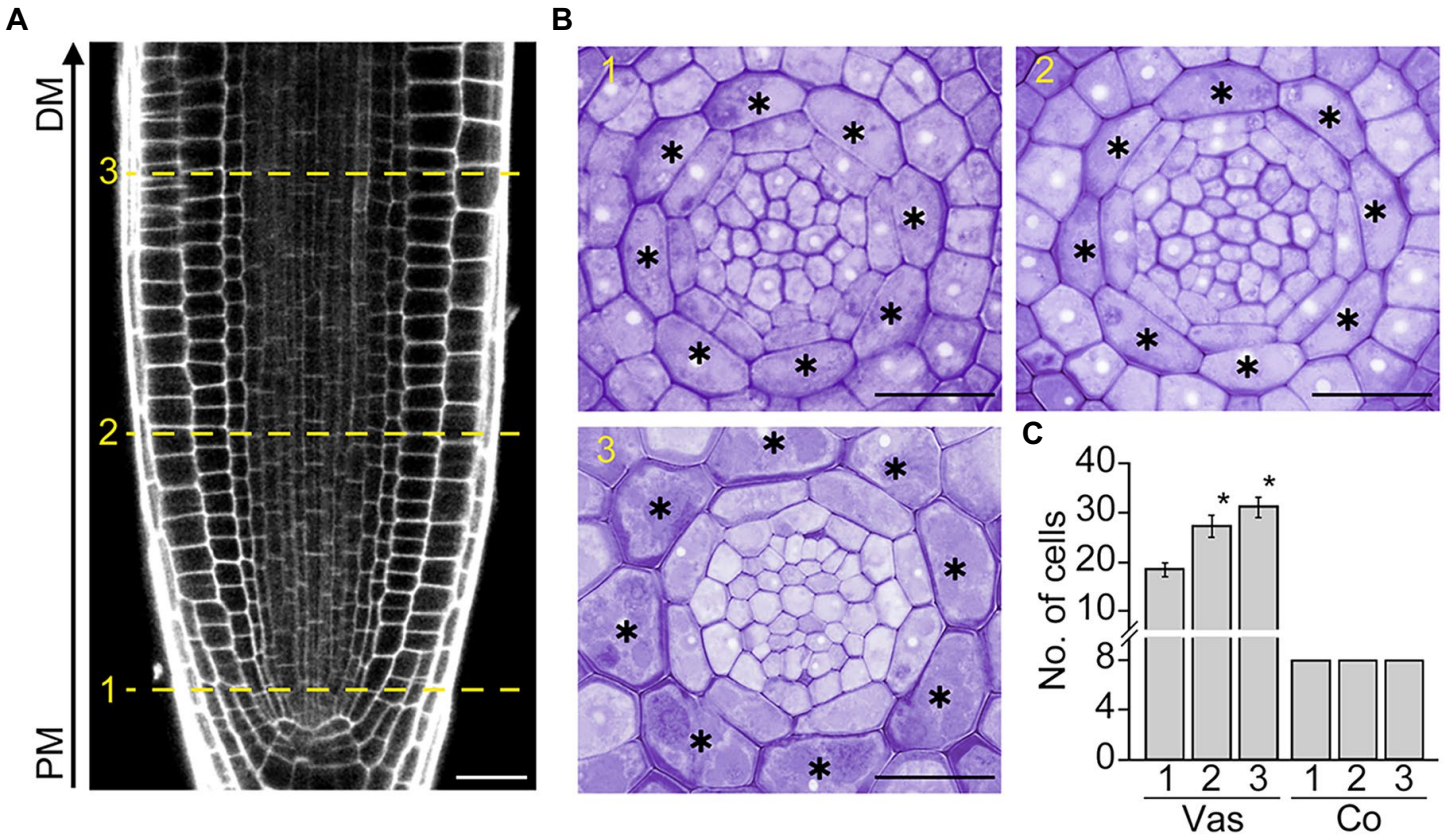
Cortex development in *Arabidopsis* roots. Longitudinal **(A)** and transverse sections **(B)** of the root apical meristem (RAM) of Col-0 wild-type plants grown in half-strength Murashige and Skoog (1/2 MS) medium for 7 days. The numbers 1, 2, and 3 indicate the positions where transverse sectioning was performed, and asterisks indicate cortical cells. PM, proximal region of the RAM; DM, distal region of the RAM. Scale bars = 20 μm. **(C)** Quantification of vascular (Vas) and cortical cells (Co) in these plants (*n* > 15). Error bars represent SD. Asterisks indicate statistically significant differences between the corresponding samples and the control (PM1; *p* < 0.01, two-tailed *t*-test).

In *Arabidopsis* roots, the development of the cortex, a single cell layer between the epidermis and endodermis, begins with the asymmetric division of the daughter cell of the cortex/endodermis initial in the root apical meristem (RAM). To further explore cortex development, we tracked changes in the number of cortical cells along the RAM, where cells divide and produce daughter cells that give rise to differentiated cells with specialized functions (Figures 1A, B). As expected, vascular cells divided in conjunction with RAM growth. The number of vascular cells was higher in the distal versus proximal region of the RAM. However, unlike vascular cells, no change was observed in the number of cortical cells: exactly eight cortical cells were present in both the proximal and distal regions of the RAM (Figure 1C). This result indicates that cortical cells do not divide in the RAM, as confirmed by the finding that the number of cortical cells in the root maturation region is identical to that of the RAM (eight cells; Supplementary Figure 1).

### Cortical auxin accumulation by NPA promotes cortical cell division

**Figure 2:**
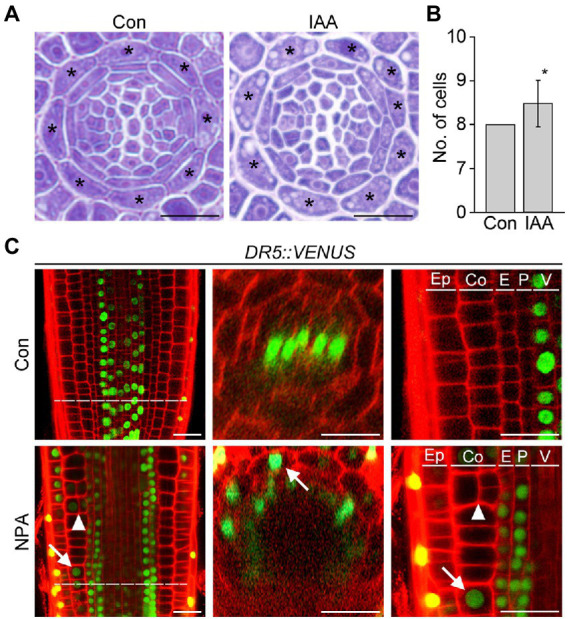
Auxin affects cortical cell division. Transverse sections **(A)** and quantification of the number of cortical cells **(B)** in the roots of wild-type plants grown under control conditions (Con) and plants treated with auxin (IAA, 500 nM) for 7 days (*n* > 15). Asterisks in the images indicate cortical cells. Error bar represents SD, and the asterisk in the graph indicates a statistically significant difference between the corresponding samples and the control (Con; *p* < 0.01, two-tailed *t*-test). **(C)** Fluorescence images of *DR5::VENUS* roots grown under control (Con, top) and 3 μM NPA-treated conditions (NPA, bottom) for 7 days (left, longitudinal sections; middle, cross-sections; right, high-magnification images of longitudinal sections). Green and red fluorescence correspond to VENUS and propidium iodide (PI) signals, respectively. Dotted lines indicate the position where cross-sectioning was performed. White arrows and triangles indicate the auxin response in cortical cells and cortical cell division, respectively. Ep, epidermis; Co, cortex; E, endodermis; P, pericycle; V, vasculature. Scale bars = 20 μm.

Auxin provides the key positional information required for cell division and differentiation, which strongly affects root cell and tissue development. To explore the possible role of auxin in cortex development, we counted the number of cortical cells in wild-type roots supplemented with auxin. Whereas untreated wild-type roots contained exactly eight cortical cells, wild-type roots treated with auxin showed a significant increase in the number of cortical cells (Figures 2A,B; Supplementary Figure 2). This result suggests that auxin is involved in the division of cortical cells.

To further investigate this notion, we analyzed changes in the auxin response and cortical cell division in response to the auxin transport inhibitor NPA using the auxin-responsive *DR5::VENUS* reporter system (Figure 2C). Previous studies suggested that auxin moves centripetally from the epidermis to the central vasculature and mediates the establishment of an auxin reflux loop (Kepinski and Leyser, 2005; Lewis et al., 2007; Baluška et al., 2010). If auxin moves centripetally, we expected that treatment with NPA would suppress centripetal auxin movement and cause auxin to accumulate in the cortex, provoking the cortical auxin response. To test this hypothesis, we visualized VENUS fluorescent signals in the roots of *DR5::VENUS* plants treated with the auxin transport inhibitor NPA. When *DR5::VENUS* plants were treated with NPA for 6 and 24 h, the VENUS fluorescent signals in the central xylem were shown to decrease gradually with increasing incubation time (Supplementary Figure 3). In addition, the number of cortical cells slightly increased in the *DR5::VENUS* plants treated with NPA for 24 h. The effect of NPA on the auxin response change and cortical cell division was more evident in the *DR5::VENUS* plants grown in NPA-treated conditions for 7 days (Figure 2C; Supplementary Figure 4). In the NPA-treated plants, the xylem-specific VENUS fluorescent signals were absent, whereas tissues surrounding the vascular tissue (including the cortex) showed VENUS signals. More importantly, the optical visualization showed that NPA induces the formation of extra cortical cells in a cortical cell file. These suggest that cortical cells divide in response to an NPA-induced cortical auxin response, and they partially support the existence of centripetal auxin movement.

**Figure 3:**
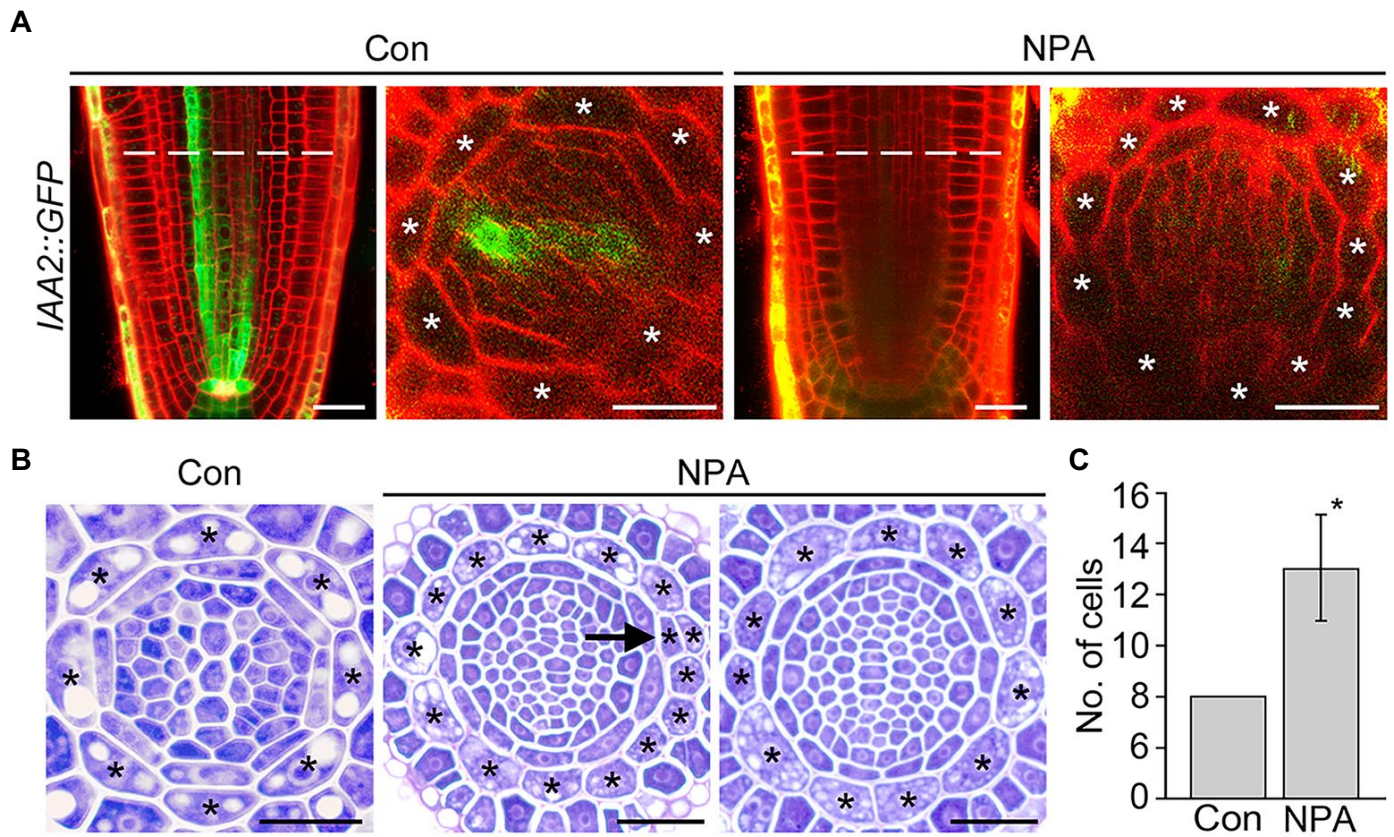
NPA strongly induces the division of cortical cells. **(A)** Fluorescence images of *IAA2::GFP* plants grown under control (Con) and 3 μM NPA-treated conditions (NPA) for 7 days (left, longitudinal sections; right, cross-sections). Dotted lines indicate the position where cross-sectioning was performed. **(B)** Transverse sections of wild-type roots were grown under control (Con) and NPA-treated conditions (NPA, 3 μM) for 7 days. Asterisks in the images indicate cortical cells, and arrow indicates periclinal division of cortical cells. Scale bars = 20 μm. **(C)** Quantification of cortical cells in these plants (*n* > 15). Error bar represents SD. The asterisk indicates statistically significant differences between the corresponding samples and the controls (*p* < 0.01, two-tailed *t*-test).

*IAA2* and *RSL4* are specifically expressed in the xylem and epidermis, and their expression is positively regulated by auxin (Yi et al., 2010; Bishopp et al., 2011). RT-qPCR assays showed that the NPA treatment reduces expression of the xylem-specific *IAA2*, but does not affect the expression of epidermis-specific *RSL4* (Supplementary Figure 5). To verify the change in the *IAA2* expression, we visualized GFP fluorescent signals in the roots of *IAA2::GFP* plants grown in NPA-untreated and treated conditions. NPA treatment strongly decreased the intensity of GFP fluorescent signals in xylem cells, but increased the number of cortical cells, supporting the visualization result of *DR5::VENUS* plants (Figure 3A). To further characterize and quantify the effect of NPA on cortical cell division, we prepared root sections from NPA-treated and untreated wild-type plants and counted the number of cortical cells by physical sectioning (Figures 3B,C). In response to NPA, wild-type plants formed approximately 13 root cortical cells due to increases in both anticlinal and periclinal divisions of cortical cells. When we quantified cell divisions in root sections, anticlinal divisions were more frequently detected than periclinal divisions (Supplementary Figure 6). In addition, we found that the cortical cell division is dependent on the concentration of NPA; the number of cortical cells increased as the NPA concentration increased (Supplementary Figure 7). Collectively, these findings indicate that the NPA-induced cortical auxin response activates divisions of cortical cells.

### Division of cortical cells in *CO2:: ΔARF5* transgenic plants

**Figure 4:**
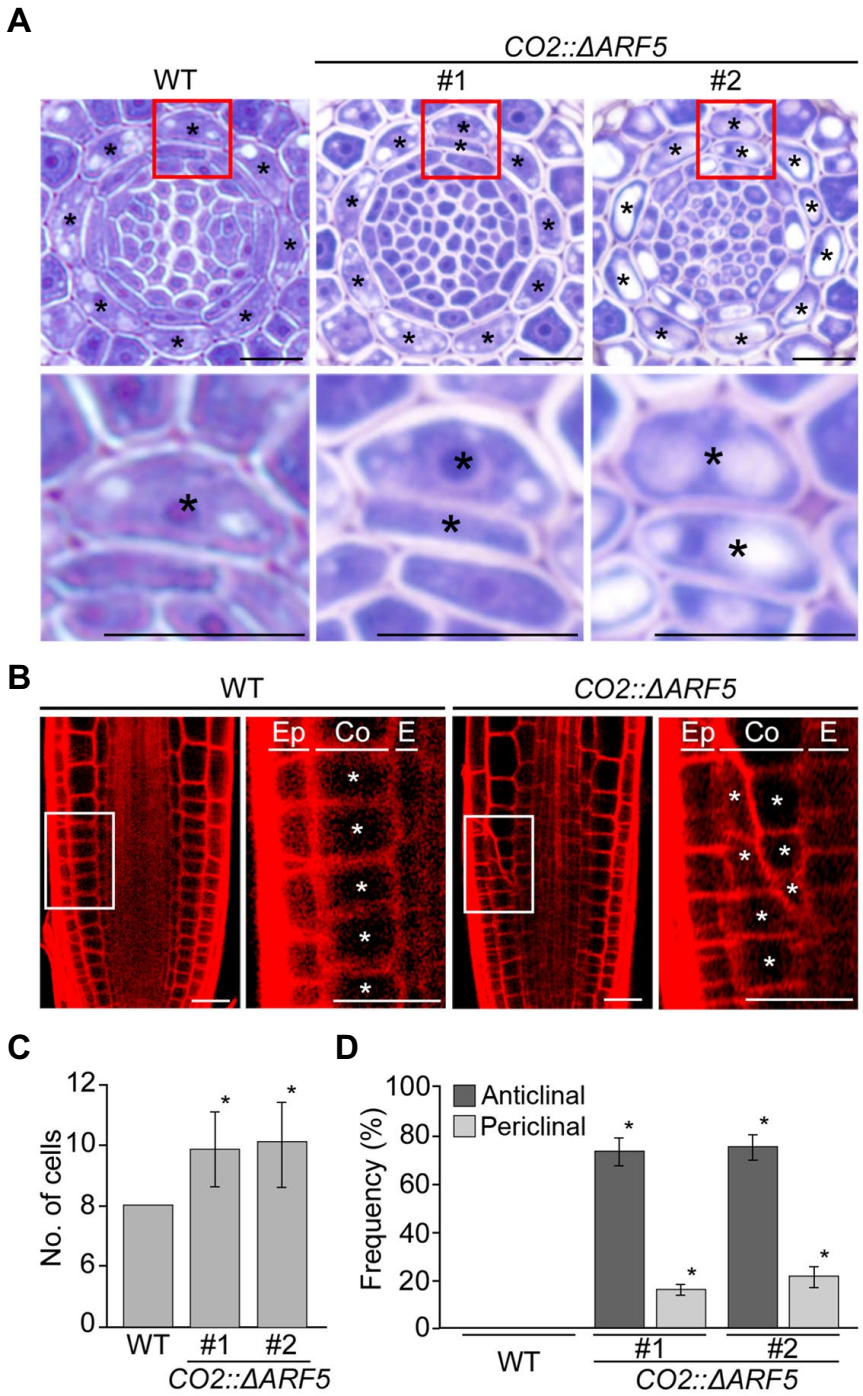
Division of cortical cells in *CO2::ΔARF5* plants. **(A)** Transverse sections of the root meristem regions of wild-type and *CO2::ΔARF5* plants grown in 1/2 MS solid medium for 7 days (top), and high-magnification images of the regions in boxes showing the periclinal division of cortical cells (bottom). #1 and 2 indicate two independent lines of *CO2::ΔARF5* transgenic plants. **(B)** Fluorescence images of propidium iodide (PI)-stained wild-type and *CO2::ΔARF5* plants (#1), and high-magnification images of the sections shown in white boxes. Ep, epidermis; Co, cortex; E, endodermis. Asterisks in **(A)** and **(B)** indicate cortical cells. Scale bars = 20 μm. **(C,D)** Quantification of cortical cells **(C)**, and the frequency of anticlinal and periclinal divisions of cortical cells **(D)** in wild-type and *CO2::ΔARF5* plants (*n* > 15). Error bars represent SD, and asterisks in the graphs indicate statistically significant differences between the corresponding samples and wild-type controls (*p* < 0.01, two-tailed *t*-test).

**Figure 5:**
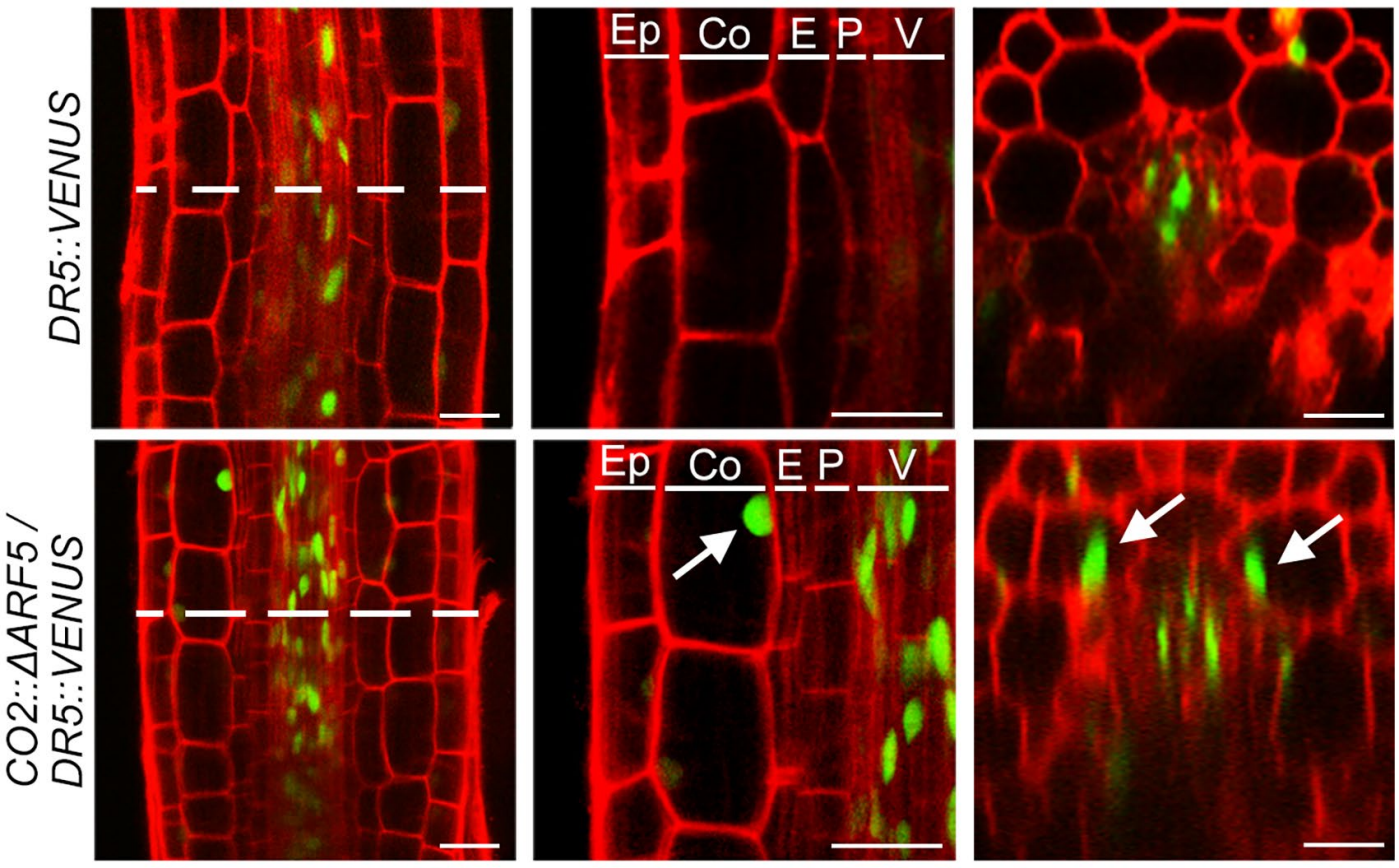
Cortical-specific auxin response in *CO2::ΔARF5* plants. Visualization of the auxin response in the roots of *DR5::VENUS* (top) and *CO2::ΔARF5*/*DR5::VENUS* plants (bottom) grown in 1/2 MS solid medium for 7 days (left, longitudinal sections; middle, high-magnification images of the longitudinal sections; right, cross-sections). White arrows indicate VENUS fluorescent signals in the cortical cells of *CO2::ΔARF5* plants. Green and red fluorescence correspond to VENUS and PI signals, respectively. Ep, epidermis; Co, cortex; E, endodermis; P, pericycle; V, vasculature. Scale bars = 20 μm.

If auxin positively regulates cortical cell division, we expected that the activation of the cortical-specific auxin response might be sufficient to provoke the division of cortical cells. To test this idea, we generated *CO2::ΔARF5* transgenic plants in which the cortical-specific *CO2* promoter drives the expression of the truncated *ARF5/MONOPTEROS* (*ΔARF5*) gene (Supplementary Figure 8). Although no obvious difference in root length and thickness was not observed between wild-type and the *CO2::ΔARF5* transgenic plants (Supplementary Figure 9), we found that *CO2* promoter-driven expression of *ΔARF5* activated cortical cell division (Figure 4). Similar to the NPA-treated wild-type plants, *CO2::ΔARF5* plants produced increased numbers of cortical cells (approximately 10 cells) due to both anticlinal and periclinal division of cortical cells, and a lower ratio of periclinal division versus anticlinal division was observed. The periclinal division of cortical cells in the *CO2::ΔARF5* plants was further observed by propidium iodide staining and optical sectioning. Unlike wild-type plants, *CO2::ΔARF5* plants displayed double cortical cell phenotype, as the NPA-treated wild-type plants did. This finding was partially supported by additional sectioning results of the root apical meristem and maturation regions in the *CO2::ΔARF5* plants (Supplementary Figure 10). Furthermore, when we visualized the auxin response in *CO2::ΔARF5* plants using the *DR5::VENUS* reporter system, *CO2::ΔARF5* plants exhibited VENUS fluorescent signals in cortical cells as well as central xylem cells (Figure 5). Collectively, these results indicate that the *CO2* promoter-mediated expression of *ΔARF5* promotes cortical cell division by inducing cortical auxin response. Taken together, these findings indicate that auxin is a key signal that determines the division of cortical cells.

### Abnormal root hair patterning in *CO2::ΔARF5* plants

**Figure 6:**
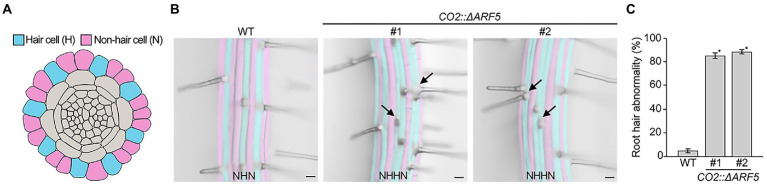
Abnormal root hair patterning in *CO2::ΔARF5* plants. **(A)** Schematic diagram of root hair patterning in *Arabidopsis* roots. Blue and pink indicate hair cells (H) and non-hair cells (N), respectively. **(B)** Root hair patterning in wild-type and *CO2::ΔARF5* transgenic plants grown in 1/2 MS solid medium for 7 days. #1 and 2 indicate two independent lines of *CO2::ΔARF5* plants. Black arrows point to the formation of double H files in *CO2::ΔARF5* plants. Scale bars = 20 μm. **(C)** Quantification of abnormal root hair patterning in these plants (*n* > 30). Error bars represent SD, and asterisks indicate a significant difference from the wild-type control (*p* < 0.01, two-tailed *t*-test).

As described above, there was no obvious difference in root length between wild-type and *CO2::ΔARF5* transgenic plants. However, when we examined root hair development in *CO2::ΔARF5* plants, the root hair pattern in these *CO2::ΔARF5* plants differed from that in wild-type plants (Figures 6A,B). In wild-type roots, hair (H) and non-hair (N) cells were regularly arranged in alternating files, with each H cell file next to N cell files. Unlike wild-type roots, *CO2::ΔARF5* roots exhibited double H cell files, in which a single H cell file was next to another H cell files. We compared the number of plants with abnormal root hair patterns, finding that the double H cell file phenotype was present in approximately 5% of wild-type plants but in 80–90% of *CO2::ΔARF5* plants (*n* > 30; Figure 6C). Similar to the *CO2::ΔARF5* plants, wild-type plants grown in NPA-treated conditions displayed the double H cell file phenotype (Supplementary Figure 11). Therefore, these findings indicate that the cortical auxin response is involved in root hair development, supporting the idea that cortical cells provide key positional information required for the determination of epidermal cell identity and patterning.

## Discussion

In this study, we revealed that cortical cells do not divide during RAM development. Instead, exogenous auxin provoked the division of cortical cells. The auxin-responsive transcription factor ARF5 contains four distinct domains: domains III and IV are responsible for interacting with Aux/IAA repressors (Weijers et al., 2006; Krogan et al., 2012). Since ΔARF5 lacks these domains, it provokes the auxin response more strongly than intact ARF5 (Krogan et al., 2012; Zhang et al., 2014). We also showed that cortical auxin response and cortical cell division are activated, when *ΔARF5* was expressed under the control of the cortex-specific *CO2* promoter. These findings indicate that auxin is a key regulator of cortical cell division.

Polar auxin transport is deeply involved in cell and tissue-specific auxin accumulation, providing key positional information required for cell division and differentiation (Friml, 2003). Polar auxin transport, which is mediated by auxin influx and efflux carrier proteins, forms directional auxin flows, such as an acropetal flow toward the root tip through the central xylem and a basipetal flow toward the root base through the epidermis (Rashotte et al., 2000; Muday and DeLong, 2001; Friml et al., 2002; Friml, 2003; Blilou et al., 2005; Michniewicz et al., 2007; van Berkel et al., 2013). Previous studies proposed that centripetal auxin flow from the epidermis to the central xylem vasculature connects acropetal and basipetal auxin flow and establishes an auxin reflux loop in the RAM (Kepinski and Leyser, 2005; Lewis et al., 2007; Baluška et al., 2010). In this study, we showed that *DR5::VENUS* plants treated with the polar auxin transport inhibitor NPA exhibit severely reduced auxin response in central xylem cells. Instead, the NPA treatment induced a cortical auxin response and cortical cell division. These NPA-induced spatial changes in the auxin response point to the existence of centripetal auxin flow and support the pivotal role of auxin in inducing cortical cell division.

In plants, the correct orientation of cell division is essential for proper embryogenesis and post-embryonic development by determining cell and tissue patterning and function; altering the orientation of cell division leads to severe defects in plant growth and development (Pietra et al., 2013; Van Norman, 2016; Luptovčiak et al., 2017; Ovečka et al., 2020). Little is known about the molecular mechanism underlying this process, but recent studies suggested that auxin is deeply involved in determining cell division patterning (Yamaguchi et al., 2013; Marhavý et al., 2016; Huang et al., 2019; Vaddepalli et al., 2021). Huang et al. (2019) demonstrated that auxin signaling regulates cell division patterning during lateral root development (Huang et al., 2019). A recent study by Vaddepalli et al. (2021) showed that the orientations of cell shape and division are tightly coupled and that auxin-dependent control of the cytoskeleton and cell shape regulates cell division orientation during embryogenesis (Vaddepalli et al., 2021). These findings suggest that auxin regulates cell division patterning, as supported by the finding that auxin is involved in the rearrangement of actin filaments and actin-dependent transport (Waller et al., 2002; Nick et al., 2009; Kovaleva et al., 2015; Zhu and Geisler, 2015).

In the current study, we demonstrated that auxin increases the number of cortical cells by promoting the anticlinal and periclinal division of cortical cells. Analysis of the frequency of anticlinal and periclinal divisions in wild-type plants treated with IAA or NPA and transgenic plants expressing *CO2::ΔARF5* showed that anticlinal division is more frequent than periclinal division in these plants (Supplementary Figure 12). Exogenous auxin treatment slightly but significantly increased the number of cortical cells due only to increased anticlinal divisions. The expression of *CO2::ΔARF5* and exogenous NPA treatment strongly increased the number of cortical cells (by approximately 2–5) by inducing both anticlinal and periclinal divisions; the frequency of anticlinal divisions was approximately 2.5-fold higher than that of periclinal divisions. When considering the tight link between the cortical auxin response and cortical cell division, these results support the previous finding that auxin plays an essential role in determining cell division patterning, and suggest that cell division orientation in the cortex is regulated by auxin.

Root hairs are formed by the differentiation of root epidermal cells (Dolan et al., 1993; Galway et al., 1994; Yi et al., 2010). In *Arabidopsis*, the epidermal cells that contact two neighboring cortical cells differentiate into root hair cells (H cells), whereas epidermal cells that contact a single cortical cell differentiate into non-hair cells (N cells). Therefore, the patterning of root hair formation in *Arabidopsis* is characterized by alternating H cell and N cell files (Dolan and Roberts, 1995; Lee and Schiefelbein, 1999). Previous many studies have suggested that cortical cells are deeply involved in determining root hair cell identity and patterning (Kwak et al., 2005; Schiefelbein et al., 2009; Hassan et al., 2010; Grebe, 2012; Song et al., 2019). In this study, we observed that *CO2::ΔARF5* plants displayed abnormal root hair patterning. Unlike the root hair patterning in wild-type plants, the alternation between H and N cell files was perturbed in *CO2::ΔARF5* plants, and the double H cell file phenotype was frequently detected. This finding suggests that the cortical auxin response and cortical cell division might be involved in epidermal cell fate. The determination of cell fate is a pivotal process in multicellular organisms. Cell-to-cell communication is one of the key processes underlying cell fate determination. Communication between cortical cells and epidermal cells mediates the determination of epidermal cell fate, and several genes involved in this process have been identified, such as *SCRAMBLED* (*SCM*), *QUIRKY* (*QKY*), and *JACKDAW* (*JKD*; Kwak et al., 2005; Hassan et al., 2010; Song et al., 2019). Although the relationship between the cortical auxin response and *SCM*, *QKY*, and *JKD* expression is largely unknown, our findings support the idea that cortical cells play a crucial role in providing information required for determining epidermal cell fate and that the cortical auxin response might be involved in this process. Further molecular and genetic analyses will expand our understanding of the mechanisms underlying this process.

## Data availability statement

The original contributions presented in the study are included in the article/Supplementary material; further inquiries can be directed to the corresponding author.

## Author contributions

GJ conceived the original screening and research plans, and agreed to serve as the author responsible for contact and ensures communication. HK, JJ, and SS performed the experiments and analyzed the data. YY and GJ wrote the article with contributions from all authors. All authors contributed to the article and approved the submitted version.

## Funding

This work was carried out with the support of the BioGreen21 Agri-Tech Innovation Program (Project No. PJ01567301) and the New Breeding Technologies Development Program (Project No. PJ01653503), Rural Development Administration, Republic of Korea. This work was also supported by the Korea Institute of Planning and Evaluation for Technology in Food, Agriculture, and Forestry through Agricultural Machinery/Equipment Localization Technology Development Program, funded by the Ministry of Agriculture, Food, and Rural Affairs (122022-03-1-HD020), and the National Research Foundation of Korea Grant funded by the Korean Government (NRF-2022R1A2C1003615).

## Conflict of interest

The authors declare that the research was conducted in the absence of any commercial or financial relationships that could be construed as a potential conflict of interest.

## Publisher’s note

All claims expressed in this article are solely those of the authors and do not necessarily represent those of their affiliated organizations, or those of the publisher, the editors and the reviewers. Any product that may be evaluated in this article, or claim that may be made by its manufacturer, is not guaranteed or endorsed by the publisher.

## Supplementary materials

The Supplementary materials for this article can be found online at: https://www.frontiersin.org/articles/10.3389/fpls.2022.953225/full#supplementary-material

## Abbreviations

RAM: Root apical meristem
NPA: *N*-1-naphthylphthalamic acid
ΔARF5: truncated AUXIN RESPONSE FACTOR 5
WT: Wild-type
CEI: Cortex/endodermis initial
IAA: Indole-3-acetic acid
CO2: Cortex-specific transcript 2
PCR: Polymerase chain reaction
RT: Reverse transcription
SD: Standard deviation
H cells: Hair cells
N cells: Non-hair cells
