# Supplementary material for: Division of cortical cells is regulated by auxin in Arabidopsis roots
Source: Front Plant Sci. 2022 Sep 14;13:953225. doi: 10.3389/fpls.2022.953225 (PMC9515965; doi:10.3389/fpls.2022.953225)
Supplement: Supplementary file 1 [file Data_Sheet_1.PDF]

Supplementary Material

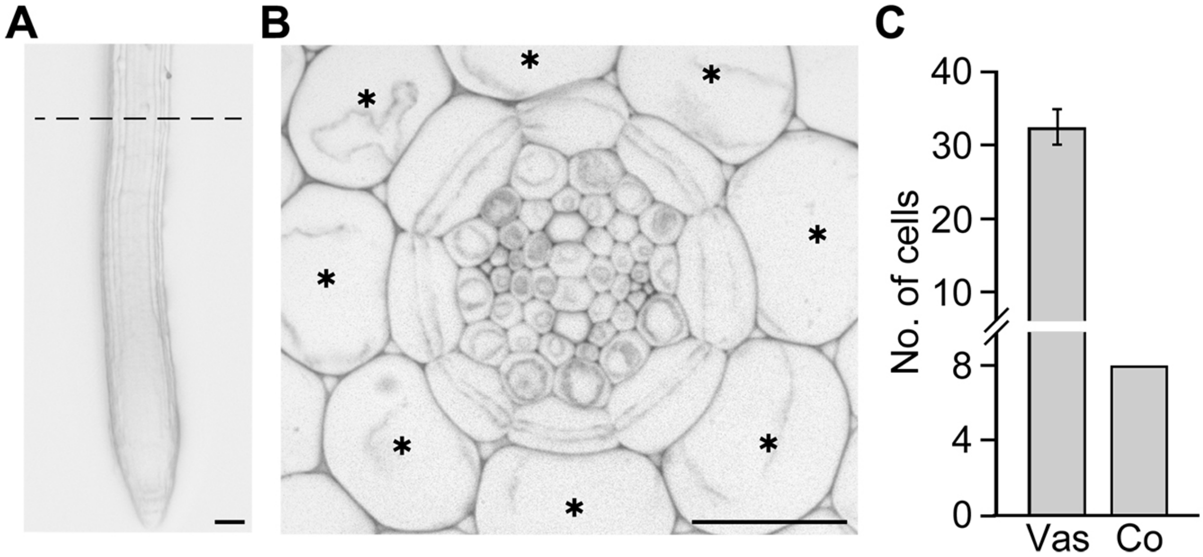

Supplementary Figure 1. Cortical cell development in the root maturation zone. **(A)** Image of the root of a Col-0 wild-type plant grown in 1/2 MS solid medium for 7 days. The dotted line indicates the position where transverse sectioning was performed. **(B)** Transverse section of the root maturation zone. Asterisks indicate cortical cells. **(C)** Quantification of vascular and cortical cells (*n*>15). Error bar in the graph represents SD. Vas, vascular cells; Co, cortical cells. Scale bars = 50 μm in (A) and 20 μm in (B).

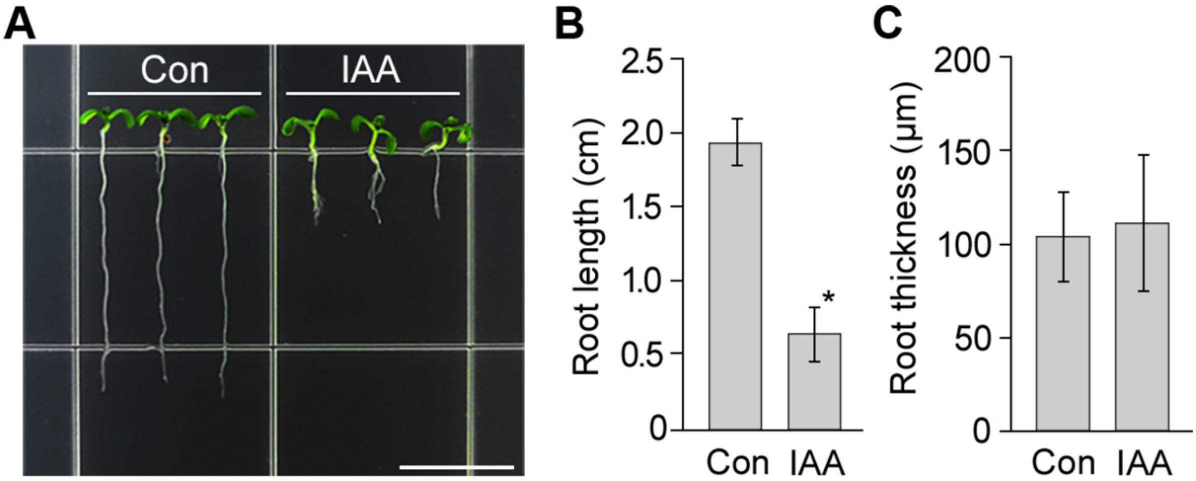

Supplementary Figure 2. Root growth in auxin-treated plants. **(A)** Root growth of wild-type plants grown in IAA-untreated (Con) and -treated (IAA, 500 nM) conditions for 7 days **(B, C)** Quantification of root length **(B)** and thickness **(C)** in these plants (n>20). Error bars represent SD. Asterisk indicates statistically significant differences between the corresponding samples and their control (P < 0.01, two-tailed t test). Scale bars = 1 cm.

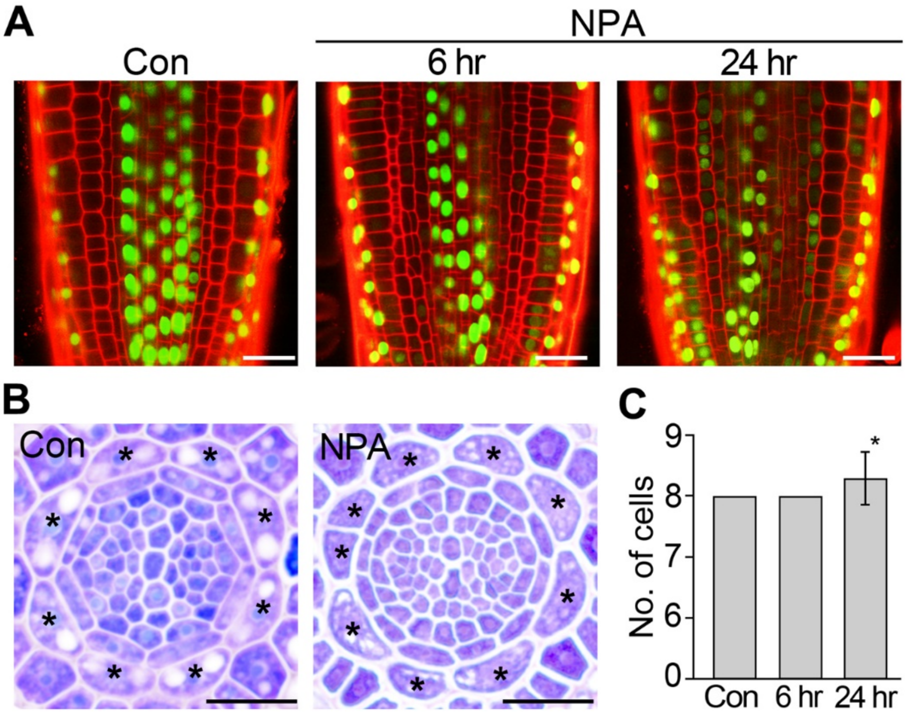

Supplementary Figure 3. Auxin response change by NPA. **(A)** Fluorescence images of NPA-treated *DR5::VENUS* roots. For NPA treatment, 7-day-old *DR5::VENUS* plants grown in 1/2 MS solid media were transferred to 3 μM NPA-containing 1/2 MS solid media, and incubated for the indicated time. Green and red fluorescence correspond to VENUS and propidium iodide signals, respectively. **(B)** Transverse sections of the *DR5::VENUS* roots grown in 3 μM NPA-untreated (Con) and -treated conditions (NPA, 24 hrs) conditions. **(C)** Quantification of cortical cells in these plants (n>20). Error bar represents SD. Asterisks in the images and the graph indicate cortical cells and statistically significant differences between the corresponding samples and their control, respectively ($P < 0.01$, two-tailed t test). Scale bars = 20 μm.

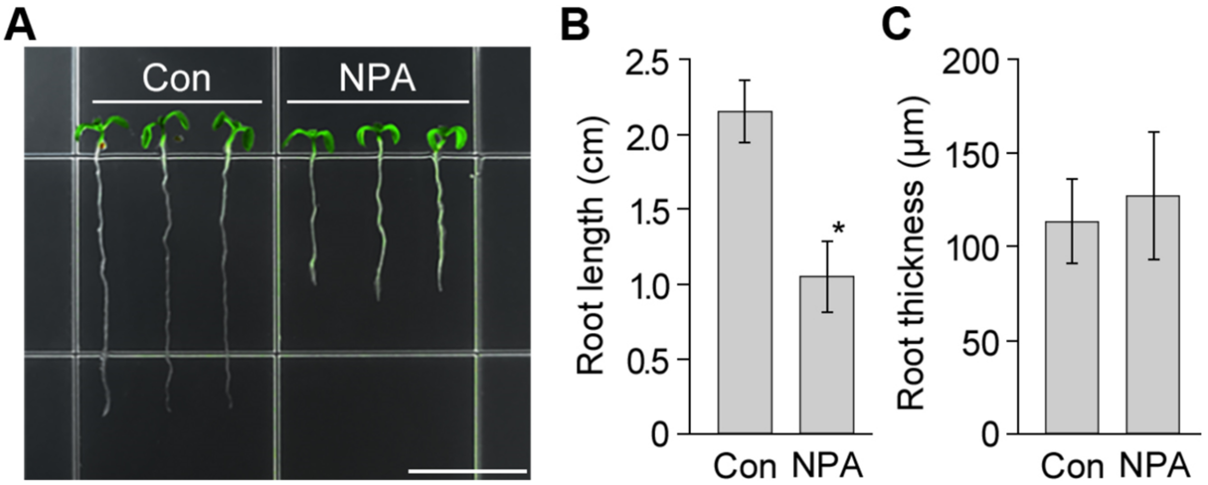

Supplementary Figure 4. Root growth in NPA-treated plants. **(A)** Root growth of *DR5::VENUS* plants grown in NPA-untreated (Con) and -treated (NPA, 3 µM) conditions for 7 days. **(B, C)** Quantification of root length **(B)** and thickness **(C)** in these plants (*n*>20). Error bars represent SD. Asterisk indicates statistically significant differences between the corresponding samples and their control ($P < 0.01$, two-tailed *t* test). Scale bars = 1 cm.

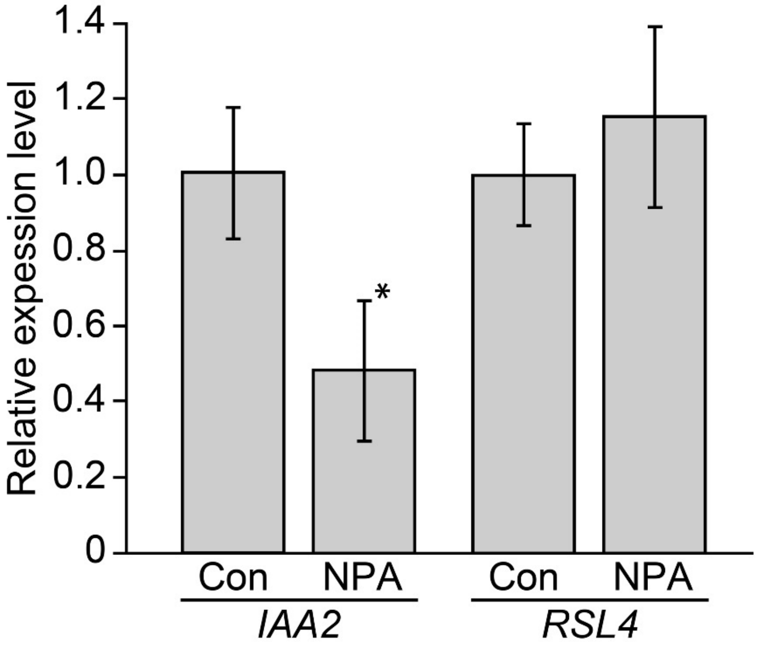

Supplementary Figure 5. Expressions of *IAA2* and *RSL4* in NPA-treated roots. RT-qPCR results showing that relative expression levels of *IAA2* and *RSL4* in wild-type plants grown NPA-untreated (Con) and -treated (NPA, 3 μM) conditions for 7 days. Error bars represent SD. Asterisk indicates statistically significant differences between the corresponding samples and their control ($P < 0.01$, two-tailed t test).

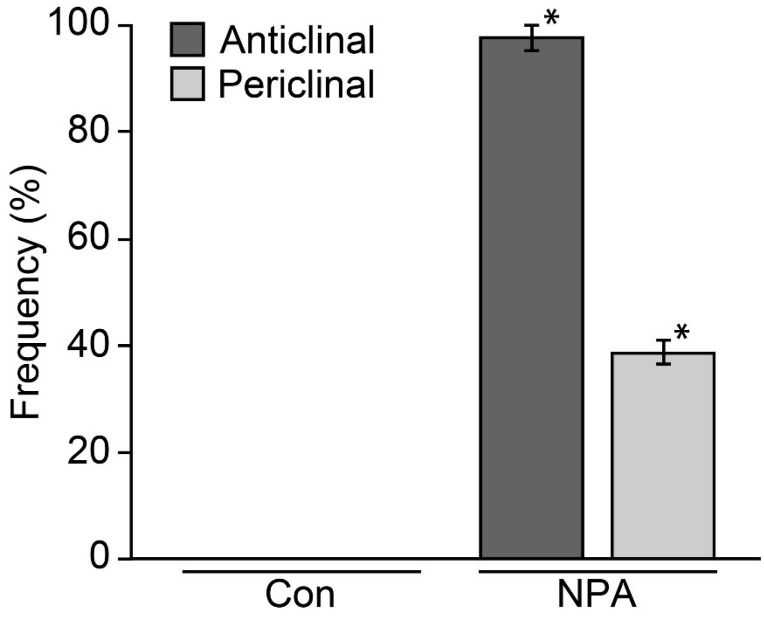

Supplementary Figure 6. Anticlinal and periclinal division of cortical cells by NPA. Quantification of the frequency of anticlinal and periclinal divisions of cortical cells in wild-type roots grown in control (Con) and NPA-treated conditions (NPA, 3 μM) for 7 days (n>15). Error bars represent SD. Asterisks indicate statistically significant differences between the corresponding samples and the wild-type control ($P < 0.01$, two-tailed t test).

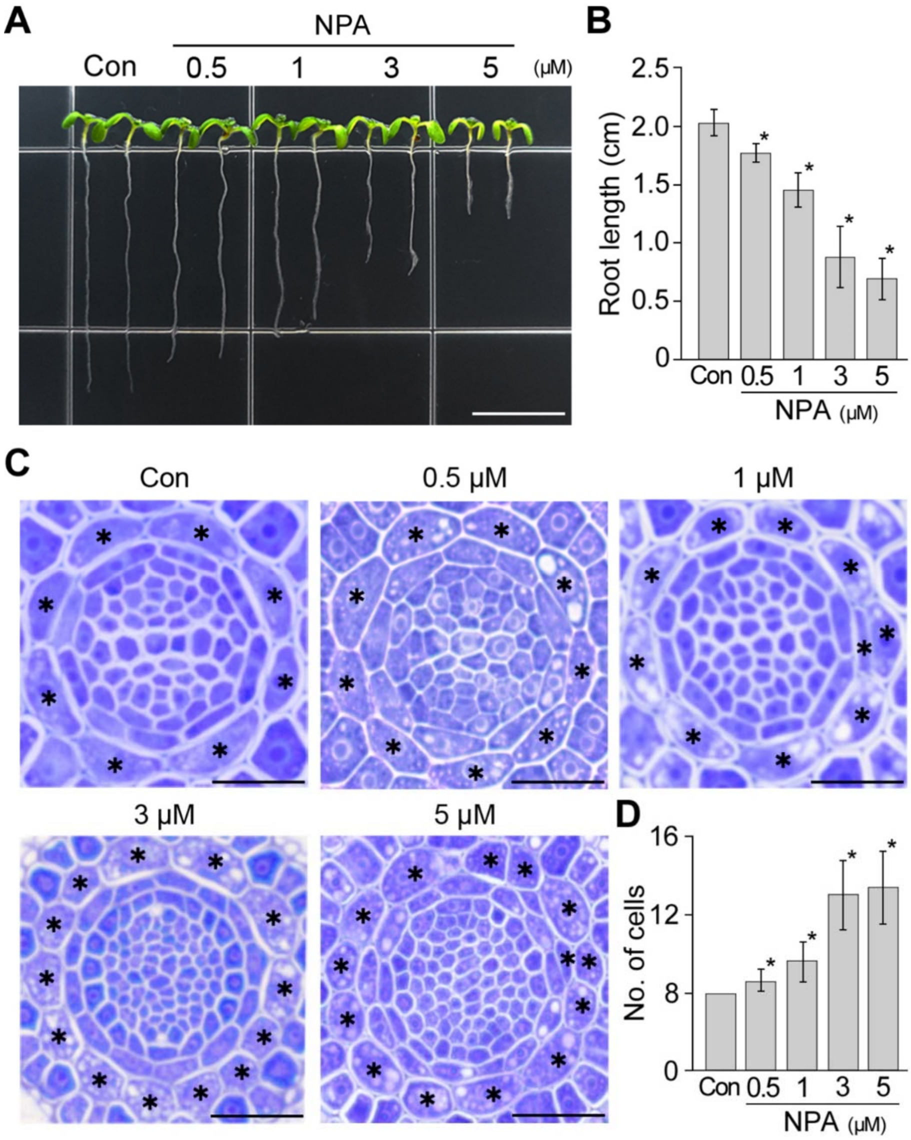

Supplementary Figure 7. Dosage-dependent effect of NPA on cortical cell division. **(A)** Root growth of wild-type plants grown 1/2 MS solid media supplemented with the indicated concentration of NPA for 7 days. **(B)** Quantification of root length in these plants (*n*>20) **(C, D)** Root sectioning images of these roots **(C)**, and quantification of cortical cells (*n*>20) **(D)**. Error bars represent SD. Asterisks on the images and the graph indicate cortical cells and statistically significant differences between the corresponding samples and their control, respectively (P < 0.01, two-tailed t test). Scale bar = 1 cm in (A) and 20 μm in (C).

A

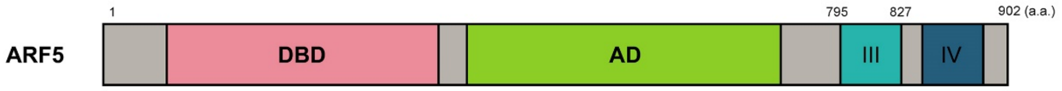

ARF5

MMASLSCVEDKMKTSCLVNGGGTITTTTSQSTLLEEMKLLKDQSGTRKPVINSELWHACAGPLVCLPQVGSLVYYFSQGHSEQVAVSTRRSATTQVPNYPNLPSQLMCQVHNVTLHADKDSDEIYAQMSL
QPVHSERDVFPVPDFGMLRGSKHPTEFFCKTLTASDTSTHGGFSVPRRAAEKLFPPLDYSAQPPTQELVVRDLHENTWTFRHIYRGQPKRHLLTTGWSLFVGSKRLRAGDSVLFIRDEKSQLMVGVRRAN
RQQTALPSSVLSADSMHIGVLAAAAHATANRTPFLIFYNPRACPAEFVIPLAKYRKAICGSQLSVGMRFGMMFETEDSGKRRYMGTIVGISDLDPLRWPGSKWRNLQVEWDEPGCNDKPTRVSPWDIETPE
SLFIFPSLTSGLKRQLHPSYFAGETEWGSLIKRPLIRVPDSANGIMPYASFPSMASEQLMKMMMRPHNNQNVPSFMSEMQQNIVMGNGGLLGDMKMQQPLMMNQKSEMVQPQNKLTVNPSASNTSGQE
QNLSQSMSAPAKPENSTLSGCSSGRVQHGLEQSMEQASQVTTSTVCNEEKVNQLLQKPGASSPVQADQCLDITHQIYQPQSDPINGFSFLETDELTSQVSSFQSLAGSYKQPFILSSQDSSAVVLPDSTNS
PLFHDVWDTQLNGLKFDQFSPLMQQDLYASQNICMSNSTTSNILDPPLSNTVLDDFCAIKDTDFQNHPSGCLVGNNNTSFAQDVQSQITSASFADSQAFSRQDFPDNSGGTGTSSSNVDFDDCSLRQNSK
GSSWQKIATPRVRT**YTKVQKTGSVGRSIDVTSFKDYEELKSAIECMF**GLEGLLTHPQS**SGWKLVYVDYESDVLLVGDDPWEEFVGCVRCIRILSPTEVQQM**SEEGMKLLNSAGINDLKTSVS

B

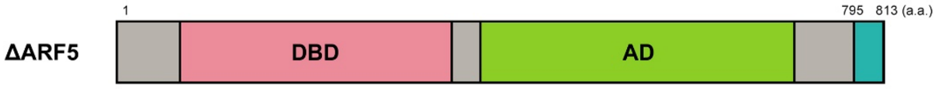

ΔARF5

MMASLSCVEDKMKTSCLVNGGGTITTTTSQSTLLEEMKLLKDQSGTRKPVINSELWHACAGPLVCLPQVGSLVYYFSQGHSEQVAVSTRRSATTQVPNYPNLPSQLMCQVHNVTLHADKDSDEIYAQMSL
QPVHSERDVFPVPDFGMLRGSKHPTEFFCKTLTASDTSTHGGFSVPRRAAEKLFPPLDYSAQPPTQELVVRDLHENTWTFRHIYRGQPKRHLLTTGWSLFVGSKRLRAGDSVLFIRDEKSQLMVGVRRAN
RQQTALPSSVLSADSMHIGVLAAAAHATANRTPFLIFYNPRACPAEFVIPLAKYRKAICGSQLSVGMRFGMMFETEDSGKRRYMGTIVGISDLDPLRWPGSKWRNLQVEWDEPGCNDKPTRVSPWDIETPE
SLFIFPSLTSGLKRQLHPSYFAGETEWGSLIKRPLIRVPDSANGIMPYASFPSMASEQLMKMMMRPHNNQNVPSFMSEMQQNIVMGNGGLLGDMKMQQPLMMNQKSEMVQPQNKLTVNPSASNTSGQE
QNLSQSMSAPAKPENSTLSGCSSGRVQHGLEQSMEQASQVTTSTVCNEEKVNQLLQKPGASSPVQADQCLDITHQIYQPQSDPINGFSFLETDELTSQVSSFQSLAGSYKQPFILSSQDSSAVVLPDSTN
SPLFHDVWDTQLNGLKFDQFSPLMQQDLYASQNICMSNSTTSNILDPPLSNTVLDDFCAIKDTDFQNHPSGCLVGNNNTSFAQDVQSQITSASFADSQAFSRQDFPDNSGGTGTSSSNVDFDDCSLRQNS
KGSSWQKIATPRVRT**YTKVQKTGSVGRSIDVTSF**

Supplementary Figure 8. Protein structure and amino acid sequence of ΔARF5. Schematic diagram of the protein structure and amino acid sequence of ARF **(A)** and ΔARF5 **(B)**. DBD, DNA-binding domain; AD, activation domain; **III**, domain **III**; IV, domain IV; a.a., amino acid. The amino acid sequence of ΔARF5 is highlighted in blue.

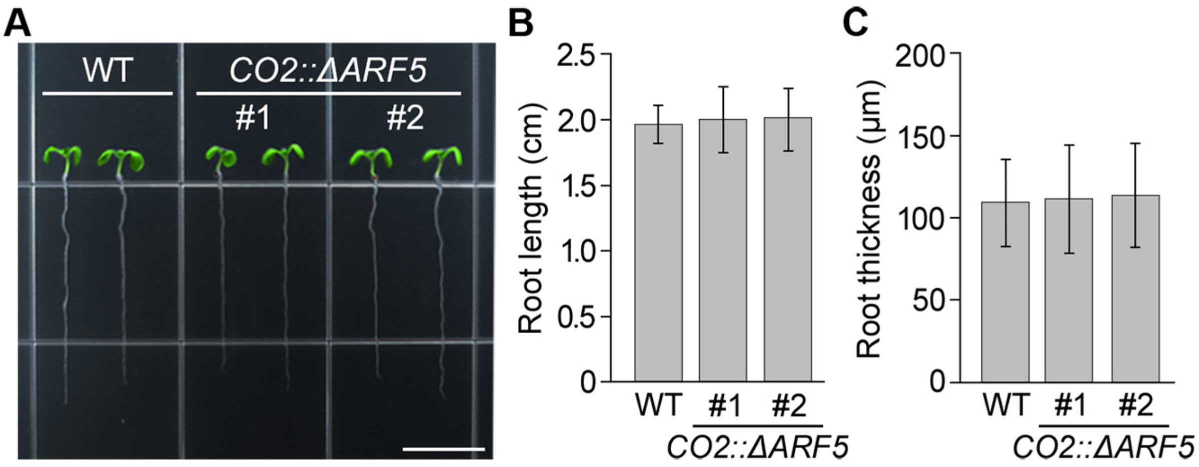

Supplementary Figure 9. Root growth in *CO2::ΔARF5* plants. **(A)** Image of wild-type and of *CO2::ΔARF5* plants grown in 1/2 MS solid media for 7 days. #1 and 2 indicate two independent lines of *CO2::ΔARF5* transgenic plants. **(B, C)** Quantification of root length **(B)** and thickness **(C)** in these plants (*n*>20). Error bars represent SD. Scale bars = 1 cm.

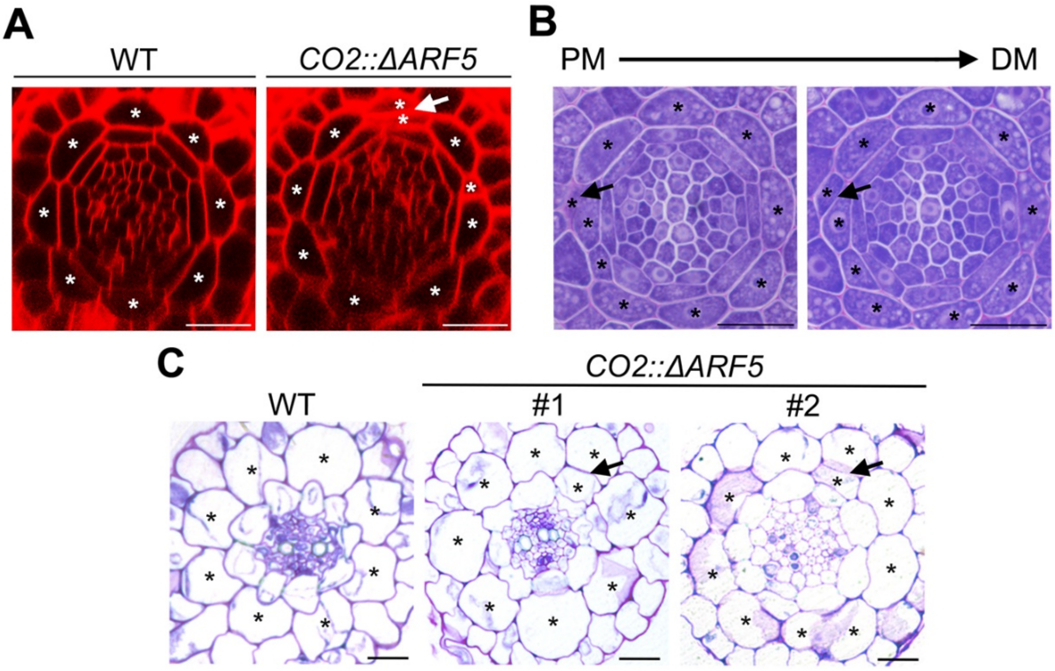

Supplementary Figure 10. Periclinal division of cortical cells in *CO2::ΔARF5* plants. **(A)** Cross-optical sections of the root apical meristem regions in wild-type and *CO2::ΔARF5* roots (#1). **(B)** A series of physical sections of the root apical meristem regions in *CO2::ΔARF5* roots (#1). PM and DM indicate proximal and distal meristem regions, respectively. **(C)** Physical sections of the maturation regions of wild-type and *CO2::ΔARF5* roots. White and black arrows point to periclinal division of cortical cells. #1 and 2 indicate two independent lines of *CO2::ΔARF5* transgenic plants. Wild-type and *CO2::ΔARF5* plants were grown in 1/2 MS solid media for 7 days. Asterisks indicate cortical cells. Scale bar = 20 μm.

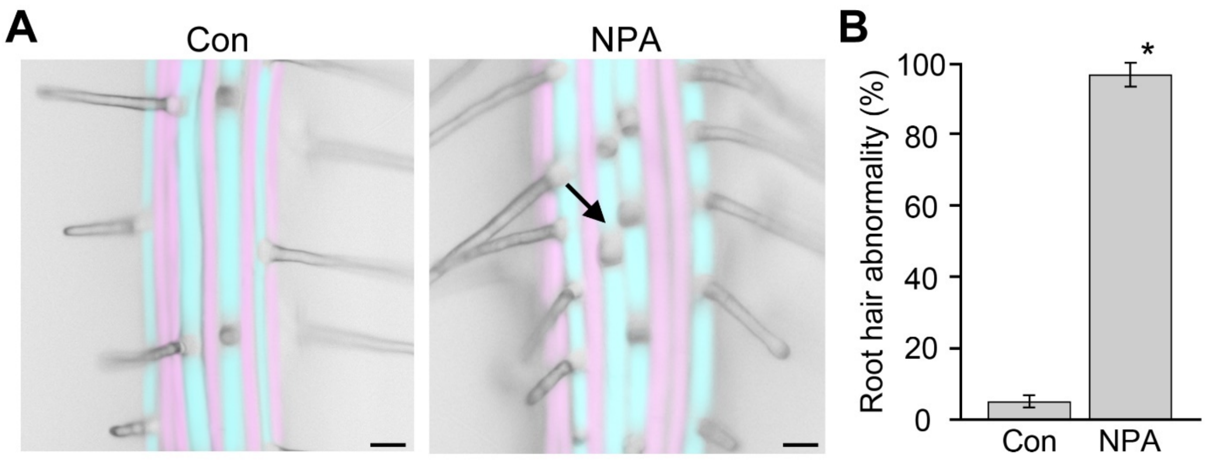

Supplementary Figure 11. Root hair patterning in NPA-treated plants. **(A)** Root hair development in wild-type plants grown NPA-untreated (Con), and -treated (NPA, 3 μM) conditions for 7 days. Blue and pink indicate hair and non-hair cells, respectively. The black arrow points to formation of double H files in the NPA-treated plants. **(B)** Quantification of abnormal root hair patterning in these plants (n>20). Error bars represent SD. Asterisk indicates statistically significant differences between the corresponding samples and their control (P < 0.01, two-tailed t test). Scale bars = 20 μm.

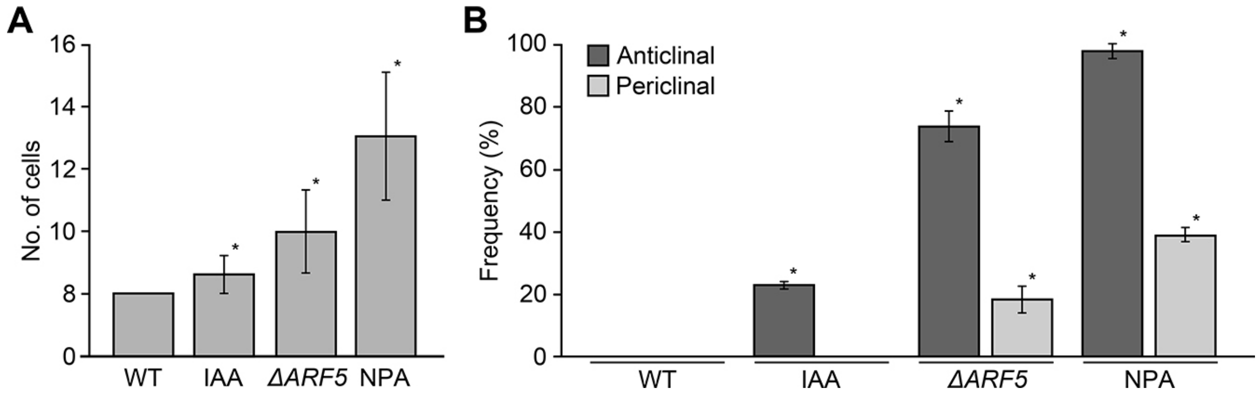

Supplementary Figure 12. Auxin regulates patterning of cortical cell division. Number of cortical cells **(A)** and frequency of anticlinal and periclinal division of cortical cells **(B)** in wild-type control (WT), 500 nM IAA-treated (IAA), 3 μM NPA-treated (NPA), and *CO2::ΔARF5* plants (*ΔARF5*) (n>15). Error bars represent SD. Asterisks indicate statistically significant differences between the corresponding samples and the wild-type controls ($P < 0.01$, two-tailed t test).

Supplementary Table 1. Primers used in this study.

Name	Sequence
CO2 pro 5 For	CGACGGCCAGTGCCAAGCTTCTAACTCCATTATTTACGACTG
CO2 pro 3 Rev	TCGAGGGGGGGCCCGGTACCGAAACTCTTGTTGCATTATTGT
IAA2 pro 5 For	GGCGGCCGCCGCGGATCCCCACATGGGTCTTCGTCTC
IAA2 pro 3 Rev	GGCCAAGCTTGATATCCCTGCTTTTGGATCAATATC
ΔARF5 cDNA 5 For	GGGGACAAGTTTGTACAAAAAAGCAGGCTATGATGGCTTCATTGTCTTG
ΔARF5 cDNA 3 Rev	GGGGACCACTTTGTACAAGAAAGCTGGGTCTAAAAACTTGTGACATCAATTG
IAA2 RT 5 For	CCATGGGACATGTTCTCTTCT
IAA2 RT 3 Rev	CATCGATCATAGTCATCATCTCC
RSL4 RT 5 For	GTTGAGCTCGGATGATCTATG
RSL4 RT 3 Rev	GACATTGTAACTTGTAAGTCC
ACT2 RT 5 For	CTTGCACCAAGCAGCATGAA
ACT2 RT 3 Rev	CCGATCCAGACACTGTACTTCCTT
